# Morphometry and Intracranial Relations of the Sphenoid Sinus in Context to Endoscopic Transnasal Transsphenoidal Surgery

**DOI:** 10.7759/cureus.40187

**Published:** 2023-06-09

**Authors:** Kusum Gandhi, Sumit T Patil, Brijesh Kumar, Manmohan Patel, Prashant Chawre, Mohtashim Ahmad, Kawal Pandita, Swapna B Parate

**Affiliations:** 1 Anatomy, All India Institute of Medical Sciences, Bhopal, Bhopal, IND; 2 Hospital Administration, All India Institute of Medical Sciences, Bhopal, Bhopal, IND; 3 Anatomy, SMBT Institute of Medical Science & Research Center, Nashik, IND

**Keywords:** maxillary nerve, vidian nerve, pituitary gland, optic nerve, internal carotid artery, endoscopy, sphenoid sinus

## Abstract

Introduction

Due to the variable degree of pneumatization, the shape and size of the sphenoid sinus are irregular. An endoscopic intranasal transsphenoidal approach is made in sphenoid sinus pathologies, sphenoid sinusitis, and sellar and parasellar diseases. A diagnostic approach to the sphenoid sinus is also done to get a better MRI scan of the pituitary. The present study aims to describe the variant types of sphenoid sinus, morphometry, anatomy, and relations of sphenoid sinus, which will be helpful to surgeons during an endoscopic approach to the sphenoid sinus.

Materials and methods

We studied 76 cadaveric sphenoid sinuses that were exposed by taking a sagittal section of 38 formalin-fixed cadaveric heads. After examining the inter-sphenoidal septum, it was removed to observe the inside aspect of the sphenoid sinus. Different dimensions of the sinus were noted down. The bulges inside the sinus due to neurovascular structures in relation to the sinus were observed.

Results

The most prominent type found was the sellar in 68.4% of cases preceded by the postsellar in 23.7% of cases. Presellar type of pneumatization was seen only in 7.9% of cases and the conchal type was absent. Intersphenoid septum was seen in 92,1% of cases, out of which 11.4% of septums were deficient on the posterior aspect. An internal carotid artery bulge in the sphenoid sinus was seen in 46% of cases. In 27.6% and 19.7% of sphenoid sinuses, bulging of the optic and vidian nerves, respectively, were seen. Some of these structures were dehiscent in the sphenoid sinus.

Conclusions

To get more space in the sphenoid sinus, the septa in the sinus are removed by surgeons, which may damage the walls of the sphenoid sinus. Knowledge of the relations of neurovascular structures with the sphenoid sinus will be helpful to surgeons during the transsphenoidal endoscopic approach to avoid any injury to these structures.

## Introduction

The paranasal sinuses are a special area of interest for surgeons working in the field of otorhinolaryngology, maxillofacial surgery, and dentistry. Paranasal sinuses are hidden inside the bones of the skull and become important due to their close relations with the eye, brain, and nose [1]. The sphenoid sinuses (SS) are two large, irregular cavities situated inside the body of the sphenoid bone, separated by a septum that usually deviates from the midline. The sphenoid sinus lies posterior to the upper part of the nasal cavity where an ostium of the sinus is seen [2]. Pneumatization of the SS starts after birth. At birth, the body of sphenoid bone consists of red marrow that is converted into yellow marrow at the age of seven months to two years. These changes are signs of the start of pneumatization which may begin before the age of two years and SS maturity may be attained by 14 years of age [3,4]. Hammer and Radberg classified the sphenoid pneumatization as conchal, presellar, and sellar depending on the extension of pneumatization around the sella turcica [5]. In the conchal or fetal type, the pneumatization of the sphenoid bone is in the anterior region and does not reach up to sella turcica. In the presellar or juvenile type, pneumatization is up to a level of sella turcica. While in the sellar or adult type, the pneumatization is below sella turcica or extends further posteriorly [6].

Due to the variable degree of pneumatization, the shape and size of the SS are irregular. Highly pneumatized SS may expose the internal carotid artery, optic nerve, and hypophysis cerebri. Other adjacent structures like the maxillary nerve, nerve of the pterygoid canal (Vidian nerve), and cavernous sinus are also very close to the sphenoid sinus cavity. On the other hand, highly pneumatized sphenoid bone provides an area to access the cranial base for surgeries of structures in close relation to the sphenoid sinus, especially the pituitary gland [7,8]. For surgical interventions in the sphenoid sinus, the transnasal and trans-septal approaches are used [9,8]. Nowadays, the endonasal endoscopic approach is becoming more popular in surgical interventions for intrasellar pathologies [10]. Chittiboina et al. used an endosphenoidal coil during transsphenoidal surgery to improve MR imaging of the pituitary gland, as MR imaging fails to detect near about 50% of microadenomas in Cushing’s disease and near about 80% of cases of dural microinvasion [11].

The endoscopic intranasal transsphenoidal approach is made in SS pathologies, sphenoid sinusitis, and sellar and parasellar diseases. A diagnostic approach to SS is also done to get a better MRI scan of the pituitary. Knowledge of the variants of SS and its relations with the internal carotid artery (ICA), optic nerve (ON), maxillary nerve (MN), and vidian nerve (VN) is important to surgeons and radiologists to avoid any injury to these structures. The present study aims to describe the variant types of SS, morphometry, anatomy, and relations of the sphenoid sinus, which will be helpful to surgeons during an endoscopic approach to the sphenoid sinus.

## Materials and methods

The study was conducted in the Department of Anatomy, All India Institue of Medical Sciences, Bhopal, India. For study purposes, 38 formalin-fixed cadaveric heads were used. All the cadavers were from Madhya Pradesh, the central part of India. Permission through letter number IHEC-LOP/2018/IM0175 from the Institutional Human Ethical Committee was taken before starting the project. The cadavers used for the study were received through a body donation program and consent for utilization of cadavers for academic and research purposes was obtained from close relatives. All the cadavers included in the study were adults, and their ages ranged between 45 and 80 years. By using a chisel, hammer, and saw, the skull cap was opened from the forehead to the occipital protuberance. The meninges and brain were removed to observe the neurovascular structures in relation to the sphenoid bone. After examination of the cavernous, it was opened to observe the ICA and MN. The mid-sagittal section of the head was taken to observe the SS. The specimens showing gross deformity and pathological changes were excluded from the study. The grossly damaged SS while taking the sagittal section was also excluded from the study. After noting the presence or absence of the intersphenoid septum of the SS, it was removed to observe inside the sinus. Probing instruments were used to explore the cavity of SS. The opening of SS was noted. The type of SS was decided according to the extent of pneumatization. The distance of SS ostium to limen nasi was recorded with the help of the vernier caliper (digital 150mm). As the shape of SS was irregular the height was measured at the anterior and posterior parts of SS and length at the upper and lower part. The cavity of SS was observed for the septa and the bulgings, which were caused due to the neurovascular structures in close relation to the sphenoid body. All the data for measurements of SS and the presence or absence of septations and bulgings of neurovascular structures were tabulated in a Microsoft Excel sheet (Microsoft Corporation, Redmond, WA). The values of mean and standard deviation and the range for dimensions of SS were calculated using Microsoft Excel. Digital photographs were captured to save the prominent features of the SS.

## Results

Ostium and pneumatization of the SS

The mean distance of the ostium was 56.33 mm from the limen nasi with a standard deviation of ± 3.41 mm. According to the degree of pneumatization of SS, it was classified into four types. The conchal type has pneumatization located in the anterior part of the SS while in the presellar, it reaches up to the anterior boundary of the sella turcica. The sellar type of sphenoid sinus has pneumatization extended to between the anterior and posterior boundary of sella turcica. In the postsellar type, pneumatization of the sphenoid sinus extended beyond the posterior boundary of sella turcica and may reach the clivus. We found no conchal type of sphenoid sinus, which may be due to the older age of cadavers. The most prominent type found was sellar in 68.4% of cases preceded by postsellar in 23.7% of cases. Presellar type of pneumatization was seen only in 7.9% of cases as shown in Table 1.

**Table 1:** The types of sphenoid sinus pneumatization

Type	Present	%
Conchal	0	0
Presellar	6	7.9
Sellar	52	68.4
Postsellar	18	23.7

Dimensions of SS

The average length of the sphenoid sinus was 21.4 ± 3.7 mm in the upper part and 26.3 ± 4.2 mm in the lower part. The average height of the sphenoid sinus was 20.1 ± 3.5 in the anterior part and 17.6 ± 2.8 mm in the posterior part (Table 2).

**Table 2:** Different dimensions of the sphenoid sinus

Measurements	Mean ± SD (mm)	Minimum- Maximum (mm)
Length (Upper)	21.4 ± 3.7	17.4 - 32.6
Length (Lower)	26.3 ± 4.2	20.3 - 35.5
Height (Anterior)	20.1 ± 3.5	16.8 - 25.5
Height (Posterior)	17.6 ± 2.8	15.7 - 21.6

Septation of the SS

**Figure 1:**
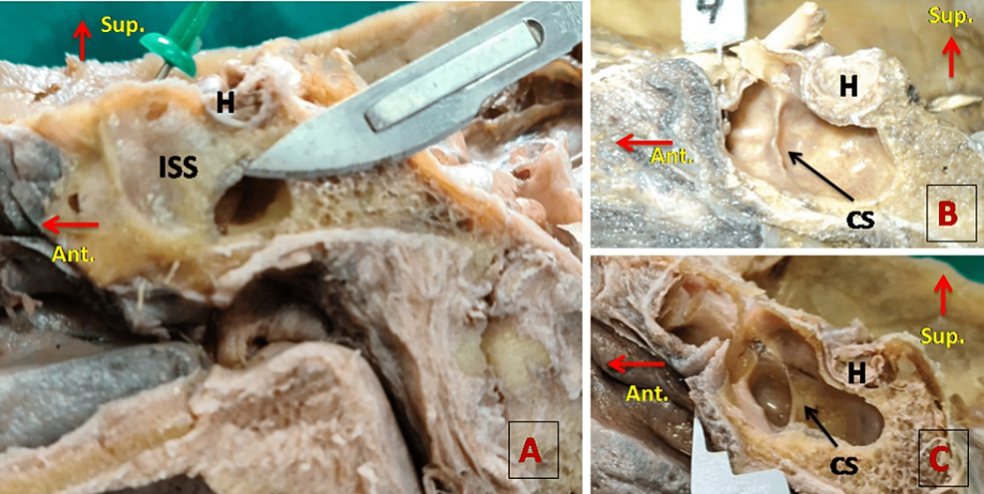
The septa of SS A: ISS having an oval opening in the posterior part. B: Small size CS. C: Well-developed CS having a concave margin, directed anteromedially. CS, crescent-shaped septum; H, hypophysis cerebri; ISS, intersphenoid sinus septum; SS, sphenoid sinus A horizontal red arrow is directed in the anterior direction and the upward red arrow shows the superior aspect of the specimen.

The intersphenoid septum was seen in 35 (92.1%) out of 38 cadavers. It was complete in 88.6% of cases extending from the anterior wall to the posterior wall. In 11.4% of cases, the intersphenoid septum was deficient on a posterior part having an oval or crescent opening as shown in Figure 1 A. We noticed one special type of septum arising from the lateral wall of SS, which was crescent-shaped, and its medial concave free margin was directed anteriorly as shown in Figure 1 (B, C). This lateral crescent septum was found in 23.7% of cases, arising just anterior to the sella turcica. Multiple septums, two to five in number, were seen in 35.5% of cases (Table 3). 

**Table 3:** Types of sphenoid sinuses

Type of septum	Septum present	Out of samples	%
Intersphenoid septum	35	38	92.1
Complete intersphenoid septum	31	35	88.6
Incomplete intersphenoid septum	4	35	11.4
Lateral crescent septum	18	76	23.7
Multiple septa (2-5)	27	76	35.5

Relations of the SS

**Figure 2:**
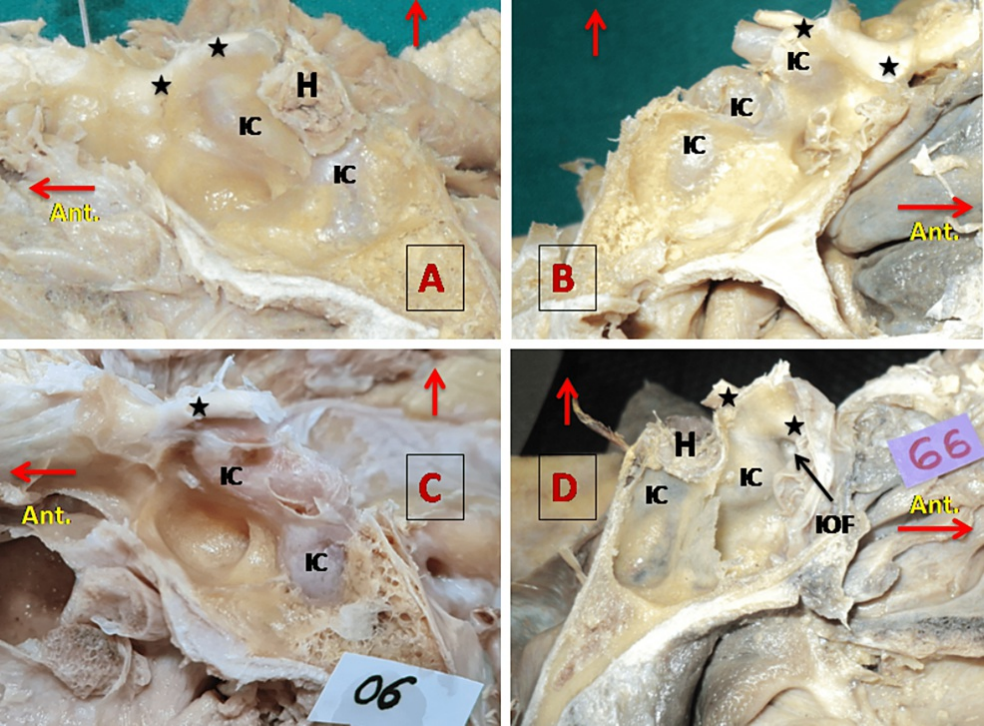
The bulges of the IC and optic nerve in the sphenoid sinus A & C: showing the lateral wall of the right SS. B & D: showing the lateral wall of the left SS. The optic nerve is marked by a black colored star '★'. A horizontal red arrow is directed in the anterior direction and the upward red arrow shows the superior aspect of the specimen. H, hypophysis cerebri; IC, internal carotid artery; IOF, infraoptic fossa; SS, sphenoid sinus.

There are many structures like the ICA, ON, VN, MN, and Onodi cells that bulge into the SS. The ICA and ON bulges have considerable clinical importance. An ICA bulge can be seen on the superolateral wall of the sphenoid sinus. In the postsellar type of sphenoid sinus, pneumatization extends on the posterior aspect, so that the ICA bulge can be seen on the posterior wall along with the superolateral wall (Figure 2 A). ICA may be separated by a thin layer of bone or completely exposed in SS (Figure 2 C). The ON bulge is present on the superolateral wall, anterior to the bulge of the ICA. At the angle of the ICA and ON bulge, there is an infraoptic fossa (IOF) as shown in Figure 2 D.

**Figure 3:**
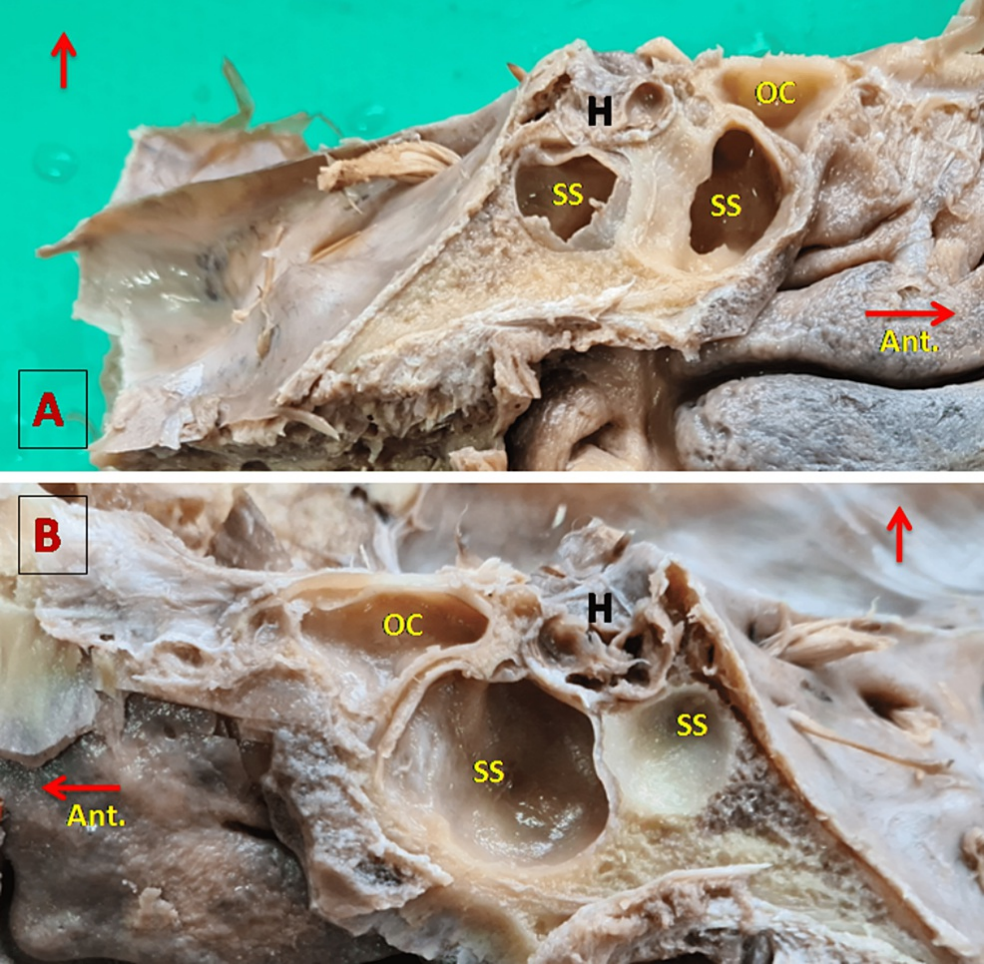
OC in the anterosuperior aspect of the SS A: Left SS, B: Right SS. A horizontal red arrow is directed in the anterior direction and the upward red arrow shows the superior aspect of the specimen. H, hypophysis cerebri; OC, Onodi cells; SS, sphenoid sinus.

**Figure 4:**
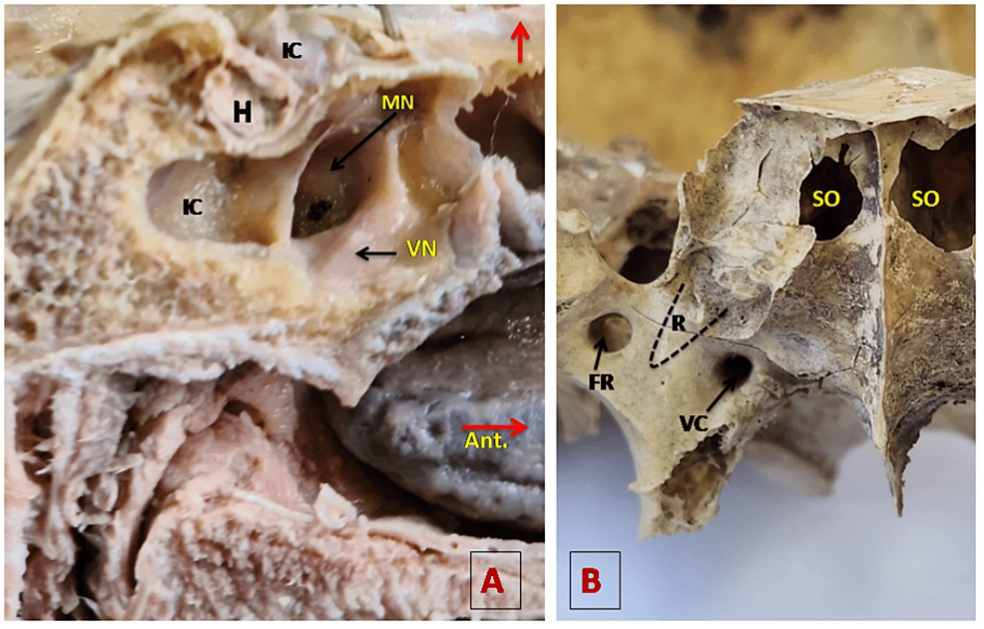
A: MN bulge and VN bulge in the anterior part of the sphenoid sinus. B: Anterior aspect of a dry sphenoid bone in which the dotted line indicates the direction of the SS recess between the FR and VC so that the bulges of MN and VN become prominent. A horizontal red arrow is directed in the anterior direction and the upward red arrow shows the superior aspect of the specimen. FR, foramen rotundum; MN, maxillary nerve; SO, sphenoid ostium; SS, sphenoid sinus; VC, vidian canal; VN, vidian nerve.

Onodi cells, which are posterior ethmoid cells, extend on the anterosuperior wall of the sphenoid sinus (Figure 3). The VN, also known as the nerve of the pterygoid canal, produces a bulge on the anterior part of the floor of the SS (Figure 4 A). The vidian canal (pterygoid canal) with VN is present on the floor of the SS while the foramen rotundum carrying MN is present on the inferolateral aspect of the SS. When the recess of SS extends between these two, the foramen rotundum and vidian canal, as shown in Figure 4, the bulge for VN and MN becomes prominent. The prominence of these different bulges on sphenoid sinus walls depends on the extent of pneumatization, and the findings of the present study are tabulated in Table 4.

**Table 4:** The relations of SS with important structures. ICA, internal carotid artery; MN, maxillary nerve; ON, optic nerve; SS, sphenoid sinus; VN, vidian nerve.

Type of bulge in SS	Present number	%	Figure no.
Superolateral ICA	27	35.5	Figure 2 D
Postero-superolateral ICA	6	7.9	Figure 2 A
Postero-superolateral ICA - exposed	2	2.6	Figure 2 C
Superolateral ON	21	27.6	Figure 2
Anteroinferior VN	15	19.7	Figure 4 A
Anterolateral MN	8	10.5	Figure 4 A
Anterosuperior Onodi cell	6	7.9	Figure 3

## Discussion

The sphenoid ostium is a natural entry point to get access to the SS. Many authors described endoscopic landmarks for the identification of the sphenoid ostium [12,13]. Most of the studies reported the presence of the sphenoid ostium in the middle of the anterior wall of the sphenoid sinus [14,15]. In earlier studies, the anterior nasal spine was used to measure the distance of the sphenoid ostium [16]. However, during the endoscopic approach to SS, the limen nasi is considered a more suitable and practical landmark [17]. In our study, we found the average distance of the ostium of SS from the limen nasi to be 56.33 ± 3.41 mm, which is remarkably comparable with the values of 56.5 mm and 55.1 mm reported by Kim et al. [17] and Gupta et al. [10], respectively.

The pneumatization type of SS is based on the location of the posterior wall of the sinus in relation to the sella turcica. Some authors classified it into three types - conchal, presellar, and sellar [9]. Another classification for sphenoid pneumatization had four types - conchal, presellar, sellar, and postsellar [18]. Later classification is more focused on the sellar region, and it is more convenient for trans-sphenoidal surgical approaches [19]. In this study, we classified the SS into four types, as it is also useful to understand the relations of important structures. In the postsellar type of pneumatization, we found the ICA bulge extending from the posterolateral aspect to the anterolateral wall superiorly. In this type, an s-shaped internal carotid artery siphon bulge was found. Anusha et al. reported that the sellar type (93%) was the most frequent, followed by the presellar (6.7%) and conchal (0.3%) [19]. Orhan et al. reported 77.4% sellar, 21.5% presellar, and 1% conchal types of SS [20]. We found 68.4% sellar, 23.7% postsellar, 7.9 % presellar, and no conchal type of SS. 

The intersphenoid septum is considered to be an important structure for transsphenoidal surgery, as it must be removed to expose the sella floor, and it may be attached to important structures like the ON and ICA, which increases the risk of damage to these structures [21]. Famurewa et al. reported at least one incomplete septum in 48.1% of cases and 50.6% of patients with multiple septa [21]. In contrast, we found 11.4% of incomplete intersphenoid septum and multiple septa (2-5 in number) in 35.5% of cases.

Famurewa et al. and Fasunla et al. reported an ICA bulge in 34.4% and 27.3% of cases, respectively [21,22]. In our study, we found an ICA bulge in 46% of cases, out of which, 10.5% of ICA bulges were composed of a thin membrane or thin plate of bone. Fujii et al. divided the ICA bulge into three parts - presellar, infrasellar, and retrosellar [23]. In our study, we found 10.5% of cases having an ICA bulge extending in all three segments. Onodi cells are a variant posterior cell of the ethmoid sinus, which extends to the superolateral part of the SS. We found 7.9% of Onodi cells but many studies reported its range to be 8-33.3% [24]. As the location of Onodi cells is in the anterior-superior part of the SS, the surgeon may mistake it for SS, and the unintentional entry may damage neighboring structures like the ON and ICA. The lateral pneumatization of the SS extending in a lesser wing of the sphenoid bone, particularly toward the anterior clinoid process (ACP)m produces the ON bulge in the SS. Sirikci et al. reported a significant relationship between ACP pneumatization and the presence of the ON bulge [25]. Wang et al. reported 11% ON bulges while in our study, we found 27.6% of SS with ON bulges [7]. The lateral recess of the SS toward the vidian canal and foramen rotundum exposes the VN inferomedially and the MN on the lateral wall of the SS. Thakur et al. reported 40.5% VN bulges and 6.38% MN bulges [26]. Hewaidi et al. reported 37.5% VN bulges and 13% MN bulges in the SS [27]. In this study, we found 19.7% VN bulges and 10.5% MN bulges.

This study has limitations due to a small sample size because of a shortage of cadavers. The study reported zero cases of the conchal type of SS, which may be due to the inclusion of cadavers aged above 45 years. The conchal type of pneumatization of SS is commonly seen in younger ages. In the cadaveric study, it was difficult to study the recesses of SS and the three-dimensional relations of SS, so a limited part was included in this study.

## Conclusions

We conclude that SS pneumatization had a vast variety of variations. The pneumatization of different SS recesses exposes different structures present at the base of the brain. To avoid any injury to these structures during the endoscopic transsphenoidal approach, precise knowledge of the type of pneumatization should be acquired. The classification of SS describing four varieties (conchal, presellar, sellar, postsellar) is better than the one describing three varieties, as the former can give a better idea regarding the relations of SS with the structures present at the base of the brain.
